# Supplementary material for: Interaction-driven transport of dark excitons in 2D semiconductors with phonon-mediated optical readout
Source: Nat Commun. 2023 Jun 22;14:3712. doi: 10.1038/s41467-023-39339-y (PMC10287636; doi:10.1038/s41467-023-39339-y)
Supplement: Supplementary file 1 — Supplementary Information [file 41467_2023_39339_MOESM1_ESM.pdf]

Supporting Information

Interaction-driven transport of dark excitons in 2D semiconductors with phonon-mediated optical readout

*Saroj B. Chand[1], John M. Woods[1], Jiamin Quan[1], Enrique Mejia[1], Takashi Taniguchi[2], Kenji Watanabe[3], Andrea Alù[1,4,5], Gabriele Grosso[1,5]**

1. Photonics Initiative, Advanced Science Research Center, City University of New York, New York, New York 10031, United States
2. International Center for Materials Nanoarchitectonics, National Institute for Materials Science, 1-1 Namiki, Tsukuba 305-0044, Japan
3. Research Center for Functional Materials, National Institute for Materials Science, 1-1 Namiki, Tsukuba 305-0044, Japan
4. Department of Electrical Engineering, City College of the City University of New York, New York, NY, 10031, USA
5. Physics Program, Graduate Center, City University of New York, New York, NY, 10016, USA

*Corresponding author email: ggrosso@gc.cuny.edu

Inventory of Supporting Information:

1. **Experimental setup**
2. **Sample preparation**
3. **Identification of exciton complexes in WS_2**
4. **DFT calculations of the valley phonon dispersion in WS_2**
5. **Power-dependent spectral emission**
6. **Supplementary data for dark exciton diffusion and energy landscape**
7. **Analysis of exciton diffusion**
8. **Exciton-exciton interaction and exciton density**
9. **Excitation, transport, and relaxation pathways for dark exciton**
10. **Supplementary experimental data for exciton diffusion**

1. Experimental setup

Photoluminescence and spectroscopy measurements are carried out in a home-built confocal microscope setup coupled to a closed-cycle cryostat. Photoluminescence experiments are performed by exciting the samples quasi-resonantly with the B exciton by a continuous-wave (CW) green laser (532 nm). Figure S1 shows the complete scheme of the optical setup. An XY galvanometer system (GM_1) in the excitation path is coupled to a 4f lens system to control the laser position on the sample. The setup allows for three independent detection paths able to measure either the emission intensity with free-space coupled APDs and the spectrum with a high-resolution grating (600 G/mm) spectrometer. One detection is possible through GM_1 with the detection occurring in the same position of the excitation. The lens system L_3 with a pinhole of 50 μm allows for confocal imaging with a spatial resolution of approximately 350nm. The second detection path occurs via GM_2 and allows to decouple the detection position from the excitation position as illustrated in Fig. S1b. The decoupling is possible because different angles at GM_1 and GM_2 result in different positions on the sample via the 4f system. The detection path through GM_2 is also confocal as it passes through the lens system L_3 and pinhole. Data from Fig.4b,c are collected by exciting the sample via GM_1 and collecting spectra via GM_2. The third detection path bypasses the confocal system and allows for the imaging of real space emission with a large field of view with an EMCCD. The latter detection path is used to collect the diffusion data shown in Fig.2 and Fig.3. In the detection paths, the laser reflection is removed from the collection signal with long pass filters at 550 nm.

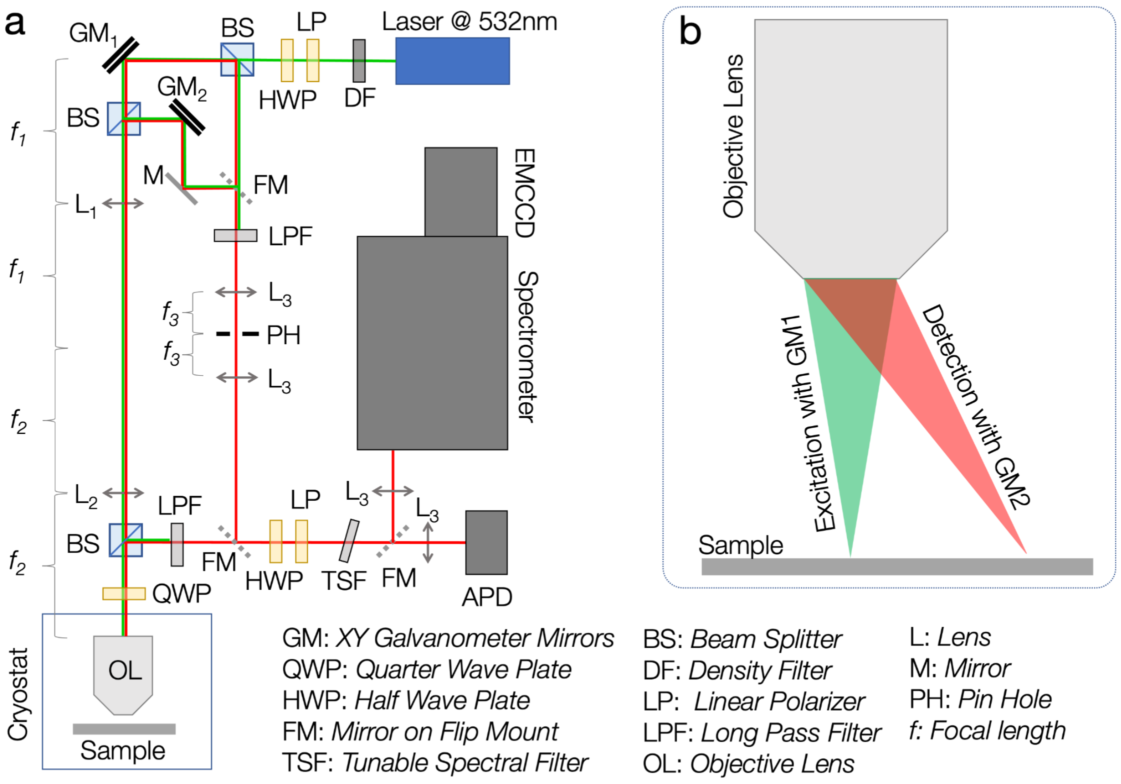

Figure S1: Sketch of the advanced high-resolution spatially-resolved PL spectroscopy setup (**a**) that allows for the decoupling of the excitation and detection (**b**).

2. Sample preparation

Figure S2 describes the steps of the sample preparation. Layers of hBN and WS_2 (from HQ Graphene) are initially exfoliated from bulk materials with a standard scotch tape technique on thin PDMS foils (X4 WF Film from Gel-Pak). The bottom hBN layer with a thickness of 20 nm is transferred on a Si/SiO_2 substrate with dry transfer using a PDMS foil and parallel engagement (step 1 in Fig. S2). The bottom hBN is then used as a substrate for WS_2 (2). The WS_2 monolayer on the PDMS foil is engaged on the bottom hBN layer using a very large angle (3). When the monolayer gets in contact with the hBN, the PDMS foil and the WS_2 are mechanically pulled in order to stretch the WS_2 before the deposition onto the hBN (4). Due to the mechanical stress of the supporting PDMS, the monolayer WS_2 is transferred with an increasing level of tensile strain. Finally, the top hBN layer of thickness 10 nm is transferred on the WS_2/hBN stack with a large angle to prevent the release of strain in the WS_2 monolayer. A microscope image of the sample and a photoluminesce map are in Figure S3. Note the PL map is taken using the collection path of GM_1 (Figure S1)

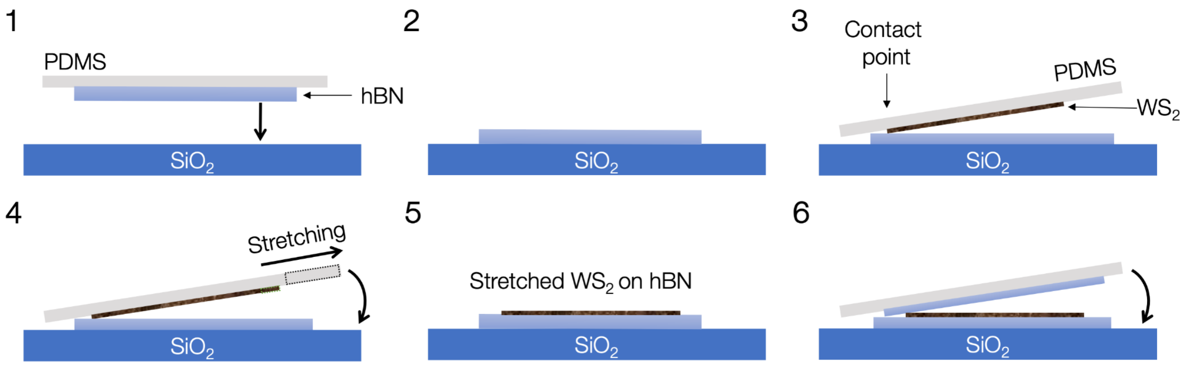

Figure S2: Illustration of the steps for the engineering of the potential energy landscape of WS_2 via strain introduced during the transfer process.

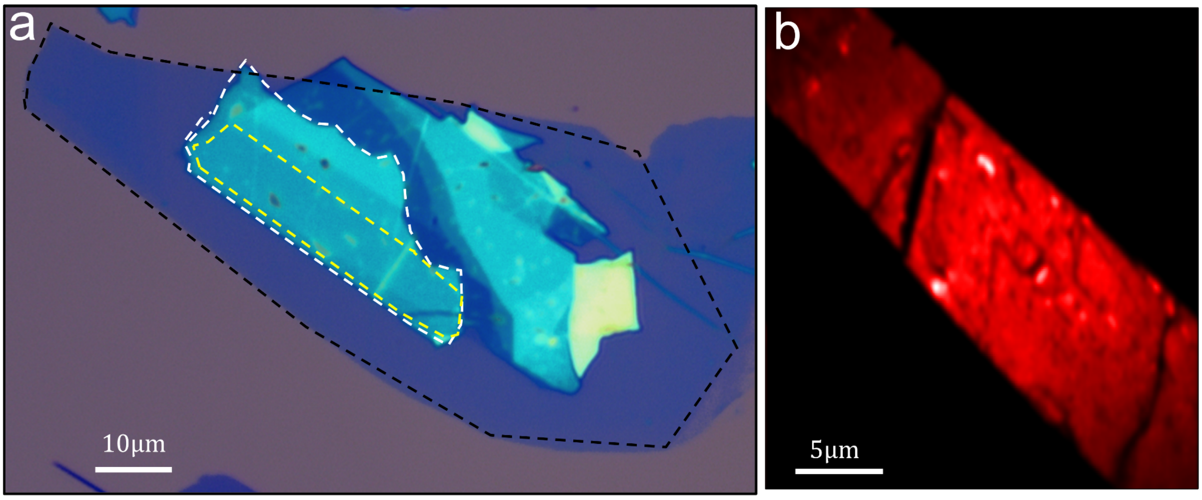

Figure S3: Microscope optical image and full PL map of the hBN encapsulated WS_2

3. Identification of exciton complexes in WS_2

The emission spectrum of encapsulated high-quality WS_2 of Fig.1b shows many bright and dark exciton peaks whose nature has been largely studied in previous works. In Figure S4a we show the emission spectra of WS_2 at T = 7K taken at a different position with respect to the one shown in Fig.1b of the main text. The identification of the peaks is consistent across the sample. Fig. S4b illustrates the multipeak fitting procedure used to deconvolute overlapping peaks and to extract their energy. Figure S4c is a zoom in the spectra range of the dark trions and phonon replicas to better visualize their peaks.

The identification of the exciton complexes is based on the comparison with the literature and corroborated by the great agreement between the energy measured here and the ones reported in Ref.[1,2] Moreover, our results are in excellent agreement with many other reports on WSe_2 whose exciton physics is very similar to the one WS_2 as the band structure is qualitatively the same.[3–5] In particular, the symmetries that govern phonon coupling and the exchange interaction, and the resulting peak order in the spectrum is basically the same except for small changes of the order of a few meV in the energy of the exciton complexes.[6,7] The absolute values and the relative difference of the energy of the exciton complexes measured in our WS_2 sample are reported in Table S1 and Table S3, respectively.

The energy detuning between the main neutral exciton X^0 and the other bright complexes (XX^0, X_T^-, X_S^-, XX^-) is in excellent agreement with other recent reports for n-doped encapsulated WS_2.[1,2] Note that at low excitation power (Fig. S4a), we observe the fine structure of the negatively charged biexcitons[1,8] confirming the high quality of our samples. At high laser power the broadening of the peaks result in the formation of one strong peak for XX^- (Fig. S7).

We measure the spin-forbidden dark exciton D^0 and the spin-forbidden dark trion D^-at 40 meV and 57 meV below the neutral exciton, respectively. Remarkably, these values are in excellent agreement with previous measurements in which the dark excitons are brightened by an in-plane magnetic field in WS_2,[2,9] and with the other reports on WSe_2. Therefore, we can assign these peaks to the dark state that are known to have an out-of-plane dipole orientation.[10,11] The intervalley momentum-forbidden dark exciton I^0 has never been reported for WS_2 to the best of our knowledge. However, this dark resonance has been reported and studied in WSe_2.[5,7,12] In WSe_2, I^0 lies in the low-energy shoulder of X_T^- and has a positive blueshift from D^0 of 10 meV due to short-range exchange interaction. In our experiments, I^0 appears clearly in the diffusion measurements of Fig.4 but it is also visible in the low excitation power spectrum in Fig.1b. In the latter, a pronounced low energy shoulder appears in the chirality plot. In our measurements, I^0 lies 6.5 meV above D^0 in good agreement with theoretical calculations.[7] The large polarization of I^0, already observed in WSe_2, further corroborates the assignment of this peak to the intervalley momentum-forbidden exciton. The weak peak at the low energy shoulder of XX^- has been recently attributed to the intervalley

momentum-forbidden dark trions T^{I}.[1] The strong peak at around 1.996 eV has been assigned to the semi-dark trion (T_1) and has been observed several times in the past in both WS_2 and WSe_2. The efficient optical recombination of D^- via the T_1 channel is made possible by the electron-electron scattering in the conduction band.[13] The low energy peaks below T_1 are the phonon replicas of the dark trion D^-. These peaks originate from the recombination of the dark states via the emission of chiral phonons. Note that phonon replicas of dark excitons have an out-of-plane emission similar to bright excitons. The attribution of these peaks to phonon replicas is confirmed by the good agreement with the theoretical calculation of the phonon energy and previous reports in both WS_2[1] and WSe_2.[3–5,7,12] In our measurements, we clearly observed the replicas of D^- due to the emission of phonons K_1, K_2, K_3, Γ_5. The phonon dispersion of WS_2 calculated by density functional theory (DFT) and their vibrational modes are shown in Figure S6. The energy of the chiral phonons in WS_2 are reported in Table S2. Note that we assign the small peak near D^-_{K2} to the Γ_5 phonon replica of D^0 as it lies at an energy 36 meV below it. However, this peak has a detuning of 43 meV with respect to I^0 and it could also be consistent with the K_3 phonon replica of I^0. Both $D^0_{\Gamma 5}$ and I^0_{K3} are allowed transitions and have been observed in WSe_2. Although the assignment of this peak does not affect the scope of this work, further investigation is needed for the correct interpretation.

In our measurements, we observe a peak appearing between D^0 and XX^- that is unlabeled in Fig.1b. The origin of this emission is unknown and requires further investigation. However, we note that a similar feature is visible in the spectra reported from WS_2[1] and WSe_2.[7]

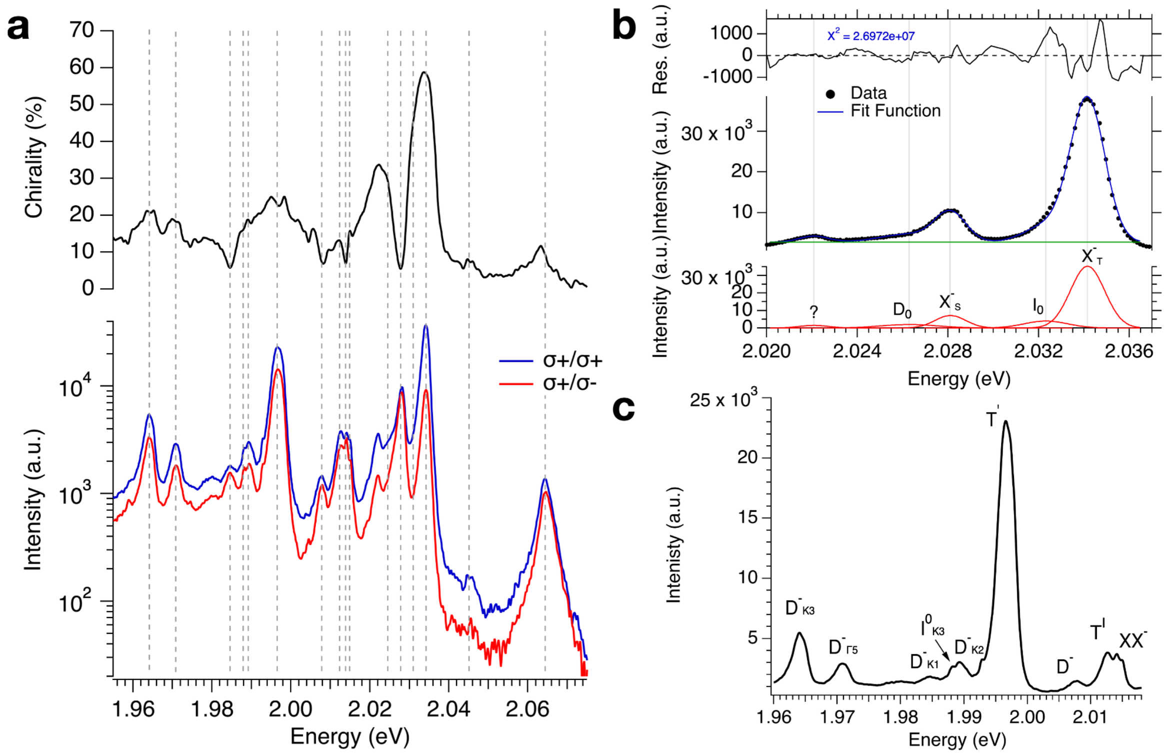

Figure S4: Supplementary data and analysis for peak identification. **a** - Emission spectra of WS_2 at T = 7K excited with σ^+circular polarization and collected with σ^+(blue line) and σ^- (red line). Top panel shows the chirality of the emission $\rho = \frac{I^+ - I^-}{I^+ + I^-}$, where I^+ and I^- is the emission intensity of σ^+ and σ^- polarized light, respectively. The peaks of bright and dark exciton complexes are highlighted by vertical dashed lines. **b** - Result of the Gaussian multi-peak fitting in the region of the spectrum of D^0 and I^0. **c** - Zoom of the spectral range that includes dark trions and phonon replicas.

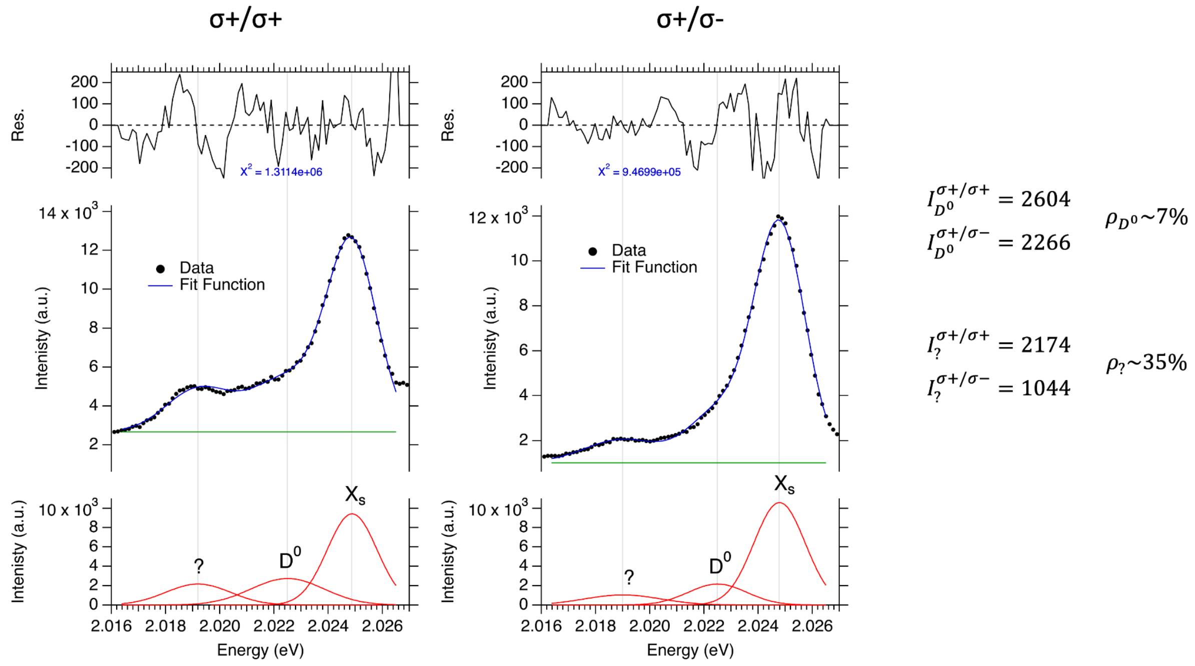

Figure S5: Degree of polarization of D^0. The deconvoluted peaks are shown for the emission spectra of WS_2 at T = 7K (shown in Figure 1 of the main text) excited with σ^+circular polarization and collected with σ^+(left panel) and σ^-(right panel). The leftmost peak is labeled with "?" as its origin is unknown. The degree of polarization $\rho = \frac{I^+ - I^-}{I^+ + I^-}$ for the deconvoluted peak of $D^0$is around 7%.

X^0	XX^0	X_T^-	I^0	X_S^-	D^0	XX^-	T^I	D^-	T_1	D_{K2}^-	$D_{\Gamma 5}^0$	D_{K1}^-	$D_{\Gamma 5}^-$	D_{K3}^-
2.0644	2.0452	2.0341	2.031	2.028	2.0246	2.0140	2.0127	2.0078	1.996	1.9893	1.9883	1.9847	1.9708	1.9641

Table S1: Energy of the exciton resonances in eV extracted from Fig.S4. An overall energy shift of 2.5 meV is observed with respect to Fig.1b. The energy difference between the exciton species is preserved in both cases.

K_2	K_1	Γ_5	K_3
16.3	22.4	37.2	42.1

Table S2: Energy of the valley phonon modes in meV calculated with DFT and extracted from Fig.S6.

	X^0	XX^0	X_T^-	I^0	X_S^-	D^0	XX^-	T^I	D^-	T_1	D_{K2}^-	$D_{\Gamma 5}^0$	D_{K1}^-	$D_{\Gamma 5}^-$	D_{K3}^-
X^0	0														
XX^0	19	0													
X_T^-	30	11	0												
I^0	33	14	3	0											
X_S^-	36	17	6	3	0										
D^0	40	21	10	6	3	0									
XX^-	50	31	20	17	14	11	0								
T^I	52	32	21	18	15	12	1	0							
D^-	57	37	26	23	20	17	6	5	0						
T_1	68	49	37	34	31	28	17	16	11	0					
D_{K2}^-	75	56	45	42	39	35	25	23	19	7	0				
$D_{\Gamma 5}^0$	76	57	46	43	40	36	26	24	20	8	1	0			
D_{K1}^-	80	61	49	46	43	40	29	28	23	12	5	4	0		
$D_{\Gamma 5}^-$	94	74	63	60	57	54	43	42	37	26	19	17	14	0	
D_{K3}^-	100	81	70	67	64	61	50	49	44	33	25	24	21	7	0

Table S3: Energy difference in meV among the exciton resonances of WS_2.

4. DFT calculations of the valley phonon dispersion in WS_2

The structural geometry relaxations and the electronic structure calculations are performed using density functional theory. We relax the WS_2 structure using ultrasoft pseudopotential with local density approximation (LDA) exchange-correlation and the plane waves implemented in the QUANTUM ESPRESSO package. The momentum space is sampled with 24x24x1 Monkhorst-Pack mesh and the kinetic energy cutoff is set to 70 Ry. After relaxation, the total force on each atom is less than 0.0001 Ry/Bohr. We used a vacuum space of 20 Å perpendicular to the WS_2 monolayer. We find an optimized lattice constant for the unstrained case of 3.121 Å. The phonon dispersion of WS_2 is calculated using the density functional

perturbation theory (DFPT) method within the Phonon package of QUANTUM ESPRESSO. We sampled momentum space with an 8x8x1 Monkhorst-Pack mesh grid.

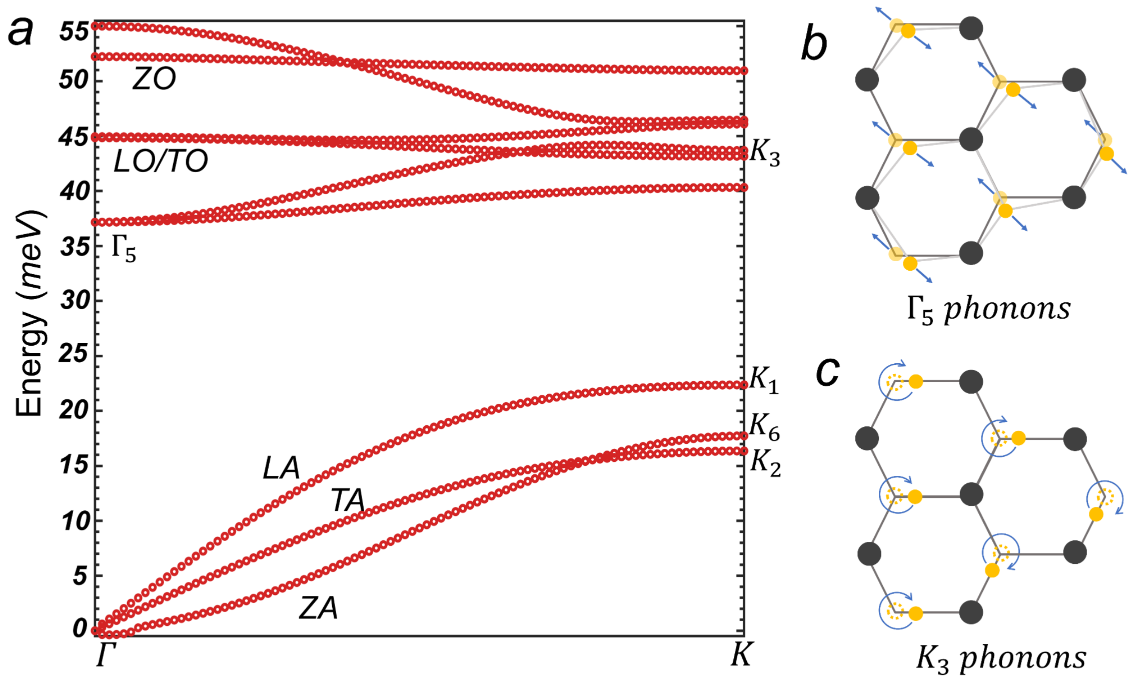

Figure S6: Phonon modes and atomic motion of valley phonon modes (a) Phonon dispersion of monolayer WS_2 along the axis between high symmetry points Γ and K. The zone center and zone-edge phonon modes are indicated. Atomic displacement of the phonon mode Γ_5 (**b**) and K_3 (**c**). The curve arrows show the in-plane circular motion of atoms around their equilibrium position indicated by faint circles.

5. Power-dependent spectral emission

Figure S7 shows the emission spectra taken with increasing laser power, from 0.05 μW to 500 μW. At high excitation power, the spectra are dominated by the strong emission of bright excitons (XX^-, X^0 and X_T^-, X_S^-), and the precise deconvolution the emission energy of D_P^- and T_1 can not be done. As previously observed in similar samples, at high carrier density a redshift of the exciton complexes occurs because of band renormalization and a reduction of the effective bandgap.[14–16] We perform the experiments with a CW laser at a wavelength of 532 nm with a chopper frequency of 500 Hz and a duty cycle of 25%. Therefore, we can exclude major effects due to heating of the sample. As far as D_P^- and T_1 are resolvable in the spectra (blue curve in Fig. S7), it is possible to observe that they experience a smaller energy shift compared to the redshift of bright exciton complexes. The redshift of bright excitons is -0.65 ± 0.05 meV, while the shift of D_P^- is -0.23 ± 0.02 meV. This difference indicates that, at high density, dark excitons undergo different interaction processes. However, we do not know how band gap renormalization affects the different exciton complexes and a quantitative analysis is impossible at this moment.

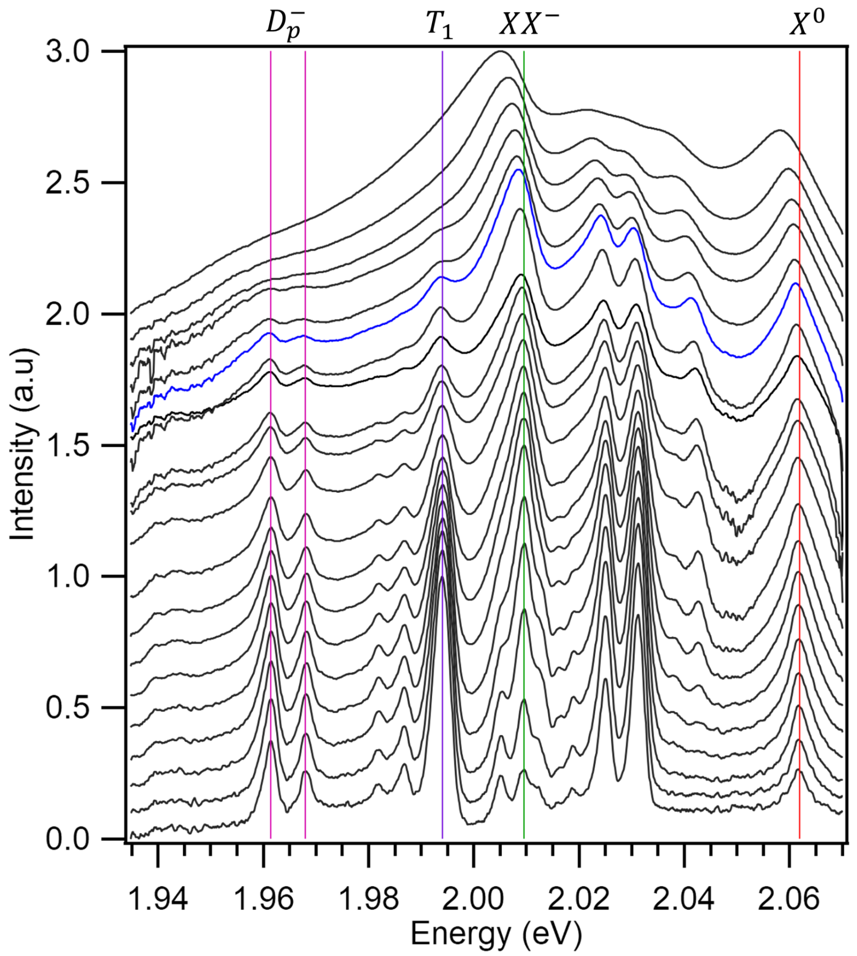

Figure S7: Emission spectra at different excitation powers ranging from 0.05 to 500μW. The emission energy at low power for the neutral excitons X^0, the negatively charged biexciton XX^-, the dark exciton T_1 and the dark trion phonon replica D_P^- is highlighted with vertical lines. The spectrum in blue color highlights the last spectrum in which dark excitons can be resolved before being inglobated in the strong emission from the bright exciton complexes.

6. Supplementary data for dark exciton diffusion and energy landscape

The expansion of the exciton cloud for the neutral exciton X^0 and the semi-dark trion T_1 as a function of the pump power is shown in Figure S8. Maps in Fig. 2, Fig. 3, and Fig. S8 are taken by using tunable optical filters to select the peak of interest. Figure S9b shows the spectrum of selected peaks X^0 (red), T_1 (blue), and $D_P^- = D_{K3}^- + D_{\Gamma 5}^-$ (pink). Figure S9a is the laser spot used in the diffusion experiments of Fig. 2, Fig. 3, and Fig. S8 measured with the EMCCD camera. Figure S10 compares the line profiles of the power-dependent emission for bright exciton and dark trion replica.

Measurements of exciton diffusion are also carried out in confocal mode in which the spatial distribution of the exciton emission is collected with APDs by exciting the system through GM1 and scanning around with GM2 to record the PL emission. Differently from the measurements performed by recording the far field emission with the EMCCD (Fig. 2, 3, S8,

S9), the confocal mode allows for better spatial resolution but lower sensitivity. Figure S11 compares the emission of the laser and the bright exciton cloud measured in confocal mode. The normalized intensity of the bright exciton overlaps with the laser profile down to 10^{-2} PL counts (see Figure S11c) and returns a diffusion length for bright excitons of $L_D = 0.8\,\mu m$.

There are some differences between the two diffusion measurements of X^0 obtained with the different imaging methods. While the EMCCD measurements have lower spatial resolution, they are highly sensitive and can detect even weak signals, such as the slow and weak decay of bright excitons, or the dark exciton emission. The APDs used in the confocal mode result in higher spatial resolution but have limited sensitivity and can mostly capture the faster and stronger decay signal of X^0.

Figure S12 illustrates the energy landscape of the sample. The area of the sample can be divided roughly in two areas. The one on the left (white dashed lines in Fig. 12a) is characterized by a linear strain gradient. The one on the right (yellow dashed lines) by a large area with very small inhomogeneity. Between these two areas there is an interface region in which strain changes quickly from 0 to ~ 0.4%. The emission spectra taken along a horizontal direction across the different regions of the sample are shown in Fig. 12c. Spectra from different positions of the "flat" region (Figure 12c) indicate a maximum variation of the bright excision energy of ~ 4 meV. We note that in the transition region where strain changes quickly, the linewidth of X^0 is much larger (7.6 ± 0.3 meV) compared to the regions with linear strain gradient or no strain (~ 3.0 ± 0.1 meV).

The raw data of Fig. 4 in the main text are reported in Figure S13. They show the normalized intensity and energy variation in the three different experiments: energy landscape, downhill diffusion, and uphill diffusion. Figure 14a displays the acquisition time as a function of distance used in the experiments performed with two galvanometers (i.e. Fig.4b,c), whereas Figure 14b shows the photoluminescence (PL) counts per second of bright excitons and charged biexcitons as a function of distance, extracted from the raw data of Fig.4c.

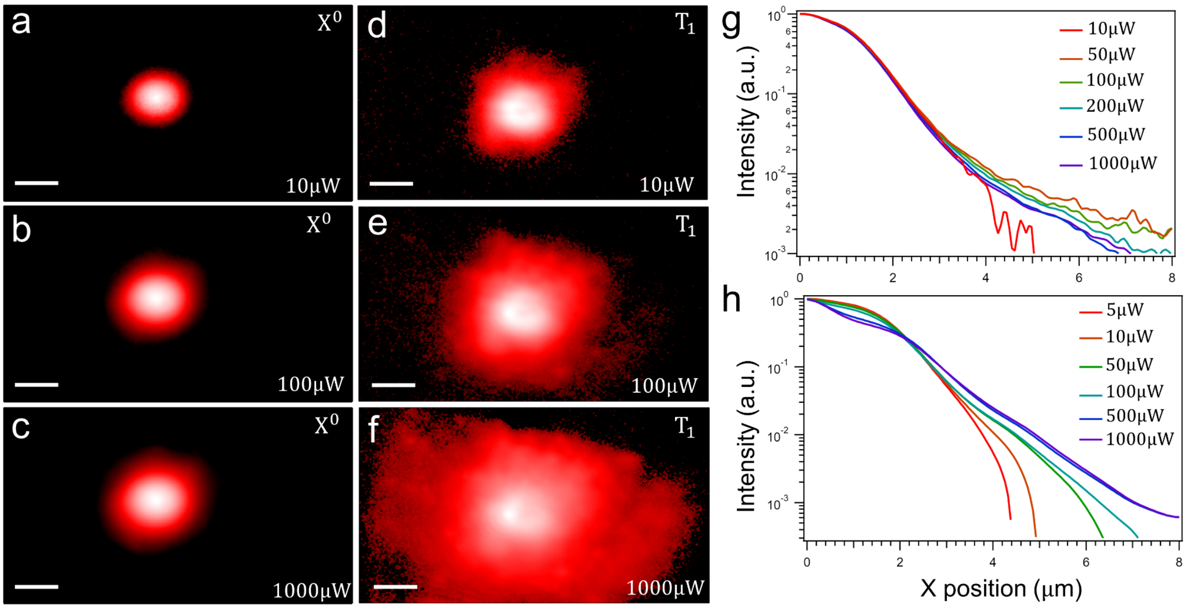

Figure S8: Power-dependent diffusion of the neutral exciton X^0 (**a-c**) and the semi-dark trion T_1 (**d-f**). The scale bar is 3μm. The horizontal emission profiles at different powers for X^0 and T_1 is shown in **g** and **h,** respectively.

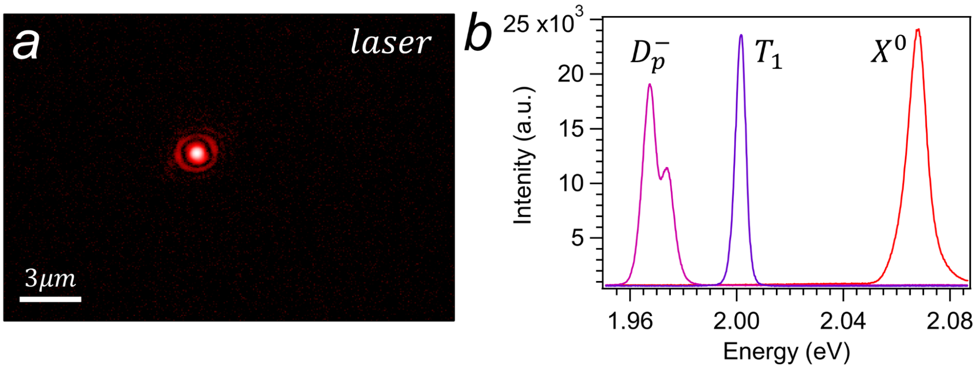

Figure S9: **a** - Reflection of the excitation laser used for the diffusion experiments. The laser has a diameter of ~2 μm. **b** - The filtered spectrum of X^0, T_1, and $D_P^- = D_{K3}^- + D_{\Gamma5}^-$ used for the spatially resolved PL emission of Fig. 2, 3 and S8.

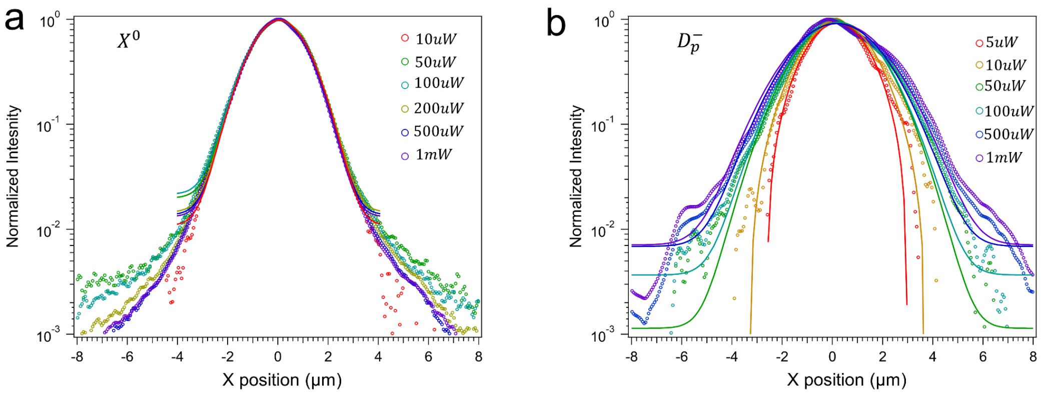

Figure S10: Gaussian fitting of line profiles from the clouds of bright excitons (X^0) and dark phonon replicas ($D_P^- = D_{K3}^- + D_{\Gamma5}^-$).

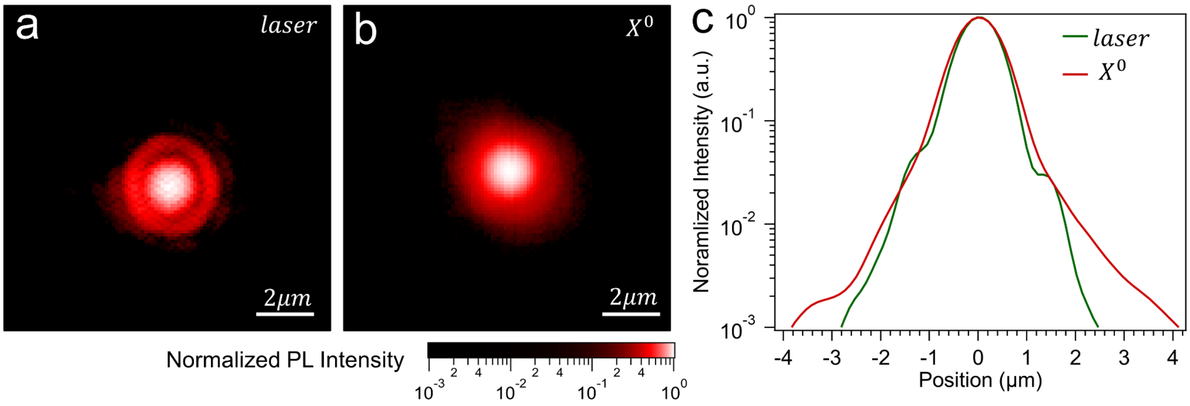

Figure S11: Diffusion measurements with confocal imaging at T = 7K. **a** - Spatial profile of the laser imaged with APDs by exciting through GM1 and scanning around with GM2 to record the reflection. **b** - Bright exciton diffusion measured in confocal mode by exciting the system through GM1 and scanning around with GM2 to record the PL emission. **c** - Comparison of the normalized intensity profile of the laser and the bright excitons emission extracted from **a** and **b.**

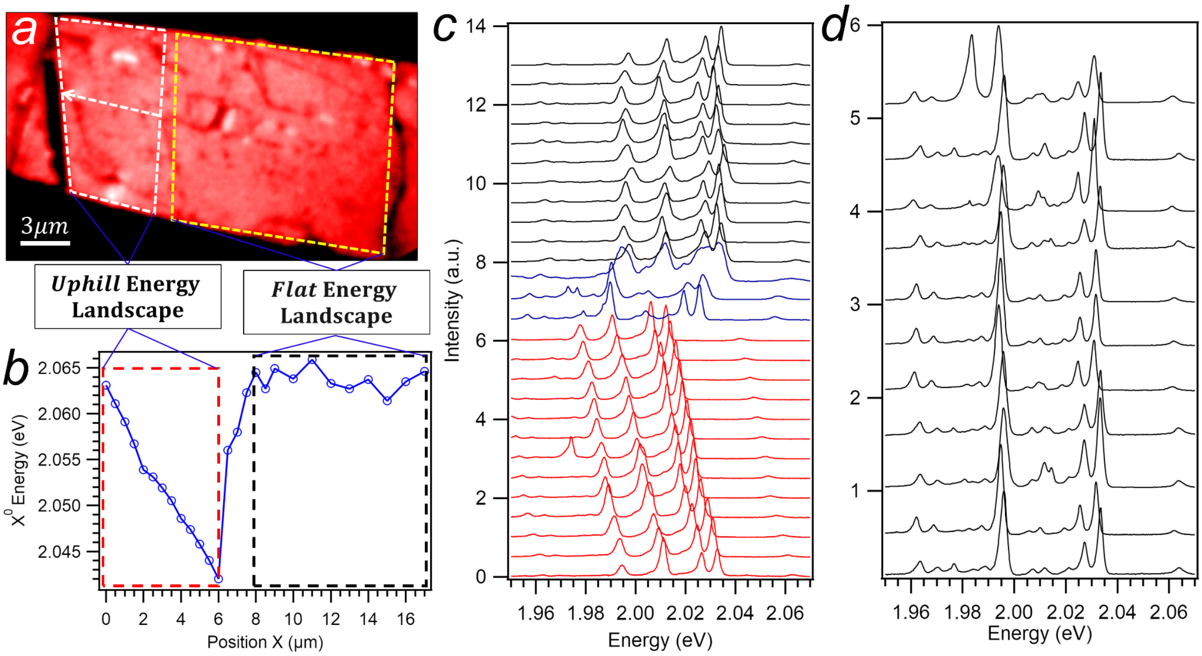

Figure S12: a - PL map of the sample highlighting regions of uphill energy landscape (white dotted box) and flat energy landscape (yellow dotted box) generated by strain variation. **b** - Energy variation of X^0 measured along a horizontal line across the uphill and flat energy landscape. **c** - Red spectra are measured in the uphill energy landscape, blue spectra are taken across the interface of the strained and unstrained region, and black spectra are taken in the flat energy landscape. **d** - Spectra taken at very low excitation powers in different positions of the region of the sample indicated by the yellow dashed rectangular box in **a**. All spectra within the yellow dotted box show a small variation in energy X^0 of ~4meV due to slight inhomogeneity, revealing a nearly flat energy landscape.

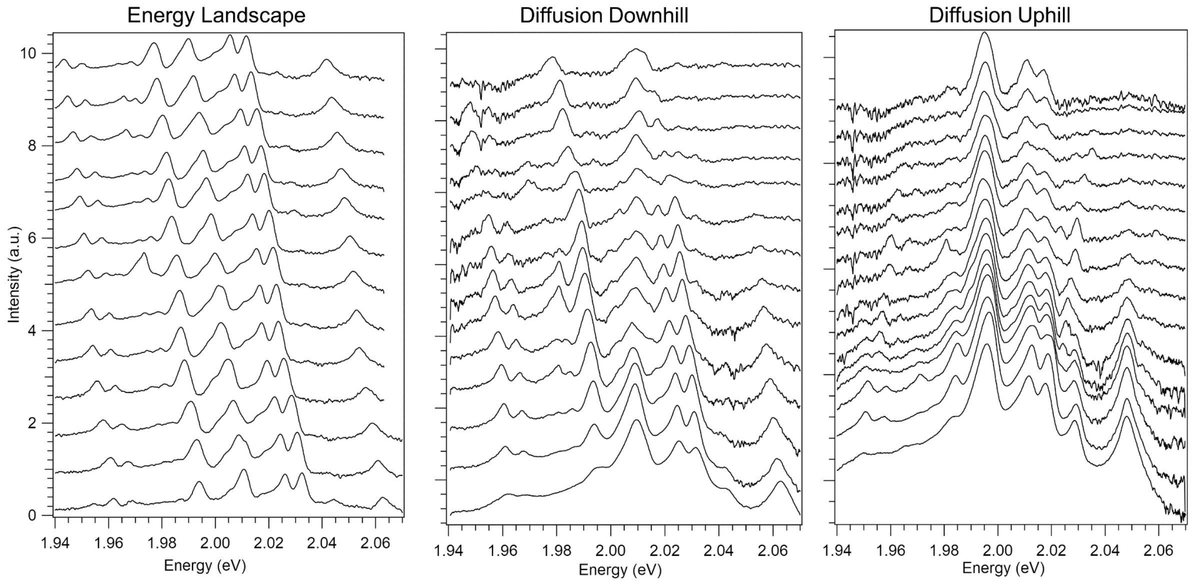

Figure S13: Spectra of exciton diffusion in engineered landscape shown in Figure 4.

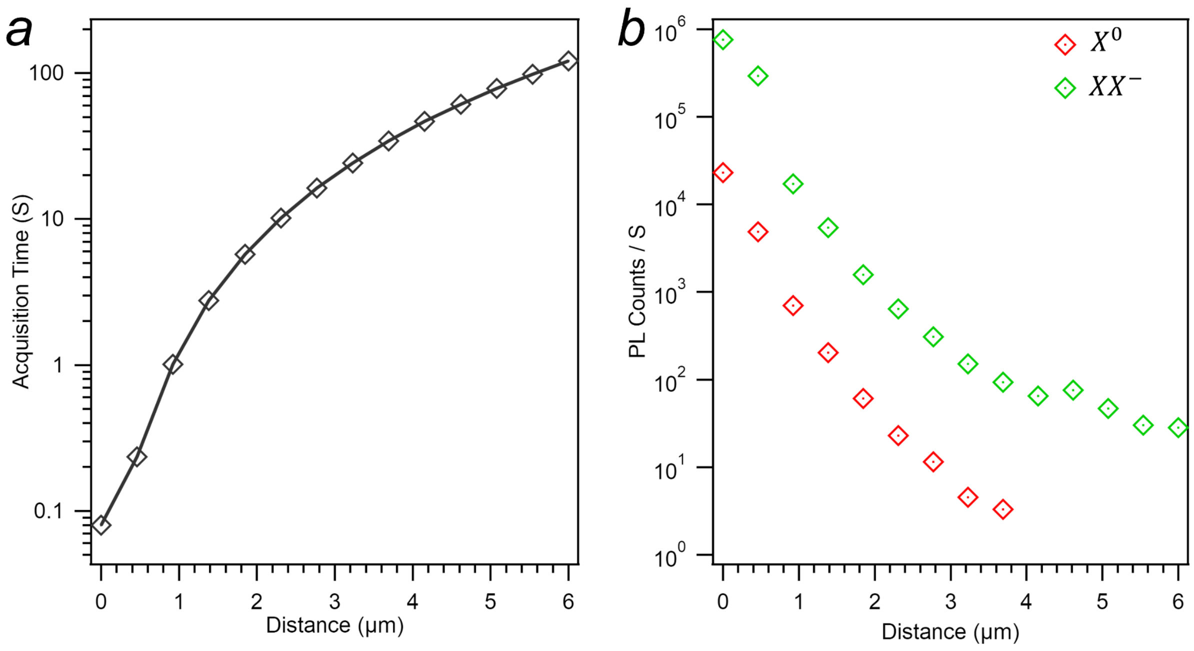

Figure S14: **a** - Acquisition time as a function of distance used in the experiments with two galvanometers. The time required for acquisition increases by three orders of magnitude as the distance from the center of the exciton increases in order to acquire PL spectra. **b** - PL counts per second of bright exciton and charge biexcitons as a function of distance as extracted from the raw data of Fig. 4c.

7. Analysis of exciton diffusion

We calculate the diffusion length L_D using a two-dimensional diffusion model with a point source that has been proven to be reliable for 2D excitons. We adopt the approach used by L. A. Jauregui et al. to analyze the emission profiles.[17] To quantify the diffusion length, we use

the asymptotic value of the steady-state density profile given by $n(r) \sim \frac{e^{-r/L_D}}{\sqrt{r/L_D}}$ to fit the emission profile of the different exciton clouds away from the laser spot. We use a steady-state width of 2.2 μm, and the extracted diffusion lengths are plotted in Figure 3f of the main text.

Previous time-dependent photoluminescence experiments have observed two mechanisms for intensity decay in TMDs: an initial rapid decay (τ_1) followed by a slower decay (τ_2). This has been observed for bright excitons [18] and interlayer excitons.[17] At this point, there are no reports of a similar behavior in the dark excitons. We note that the value of L_D measured in the steady-state and in low exciton density regime would be dominated by a slower decay rate. Furthermore, the lifetime τ of spin-dark excitons reported in the literature ranges from 110 ps to 250 ps.[4,19] For the purposes of estimation and consistency with the calculations of exciton density, we use a lifetime of 250 ps and we estimate the diffusion constant according to $D = L_D^2/\tau$. We find that the diffusion constant (D) ranges from 68 to 240 cm^2/s in the unstrained direction ($+x$), while it ranges from 40 to 110 cm^2/s in the strained uphill direction ($-x$). The diffusion constant of dark excitons increases with excitation power due to repulsive interaction among dark excitons.

8. Exciton-exciton interaction and exciton density

For small values of exciton momentum, the strength of the exchange interaction can be estimated as $U_{ex} \sim a_o^2 E_b$, where E_b is the binding energy and a_o is the Bohr radius.[20,21] The strength of exchange interactions U_{ex} of bright and dark excitons is expected to be similar as they have a similar Bohr radius (a_o) and binding energy (E_b). The Bohr radius for bright and dark excitons in WS_2 have been recently calculated by P. Li, et al. to be 2.1 nm and 1.8 nm, respectively.[22] The binding energy of bright excitons, which is in the order of 0.7 eV,[23] changes of about 10 meV in dark excitons due to different short-range electron-hole exchange interactions.[7,24] A range of values for the interaction strength of bright excitons of $7.5 \cdot 10^{-12} - 1.6 \cdot 10^{-11}\ meV\ cm^2$ can be estimated from the reported experimental values of the exciton binding energy (ranging from 0.32 to 0.7 eV) and the measured Bohr radius of 1.53 nm. [23,25,26]

The exciton density can be estimated with a model based on coupled rate equations that account for both radiative and non-radiative decay pathways. To estimate the order of magnitude of the exciton density, we consider natural bright (X^0), indirect (I^0) and dark excitons (D^0). We solve a set of coupled rate equations that take into account the energetic ordering of the exciton species ($E_{X^0} > E_{I^0} > E_{D^0}$), the phonon-mediated scattering processes among different bands, and the different lifetimes.

Upon excitation (with a generation rate of G), the bright exciton X^0 can relax towards the I^0 or D^0 state. The $X^0 \rightarrow I^0$ transition is induced by the interaction with chiral phonons K_3 which promote the spin-preserved intervalley scattering of electrons in the conduction bands from K to K'. Schematics of these interactions are shown in the Fig1e of the main text. The $X^0 \rightarrow D^0$ transition is induced by the interaction with chiral phonons Γ_5 which promote the spin-flipping intravalley scattering of electrons in the conduction bands. The transition $I^0 \rightarrow D^0$ is mediated by scattering with free electrons. An estimate for the scattering rates of these transitions is provided by recent calculations performed for WSe_2 at T=4K.[27] From Ref. [27] we extract $R_{K_3} = 2.5\, ps^{-1}$, $R_{\Gamma_5} = 1.75\, ps^{-1}$ and $R_{ID} = 2.75\, ps^{-1}$. To the best of our knowledge there are no measurements of the radiative lifetime of intervalley excitons in WS_2, so we use the values reported for WSe_2 in which the lifetimes have been measured consistently for bright, dark and intervalley excitons at cryogenic temperature.[4] We note that the lifetime in WS_2 for bright and dark excitons has the same order of magnitude as WSe_2, and we expect the same for the intervalley. From Ref. [4] we get $\gamma_{X^0} = 5\, ps$, $\gamma_{I^0} = 200\, ps$ and $\gamma_{I^0} = 250\, ps$.

In the calculations of the exciton density, we also consider nonradiative exciton-exciton annihilation (EAA) processes to include any possible Auger recombination that could happen at high exciton density. However, this process is strongly suppressed in encapsulated samples.[28] For encapsulated WS_2 monolayer, the exciton−exciton annihilation rate constant has been measured to be $C_{EAA} \sim 5 \cdot 10^{-16} cm^2 ps^{-1}$. [28,29] The effect of this annihilation process emerges only at very high density. With all these considerations, the rate equations read:

$$\dot{n}_{X^0} = G - n_{X^0}\gamma_{X^0}^{-1} - n_{X^0}(R_{K_3} + R_{\Gamma_5}) - (n_{X^0})^2 C_{EAA}$$

$$\dot{n}_{I^0} = n_{X^0} R_{K_3} - n_{I^0}\gamma_{I^0}^{-1} - n_{I^0} R_{ID} - (n_{I^0})^2 C_{EAA}$$

$$\dot{n}_{D^0} = n_{X^0} R_{\Gamma_5} + n_{I^0} R_{ID} - n_{D^0}\gamma_{D^0}^{-1} - (n_{D^0})^2 C_{EAA}$$

The results of the calculations are shown in Figure S15.

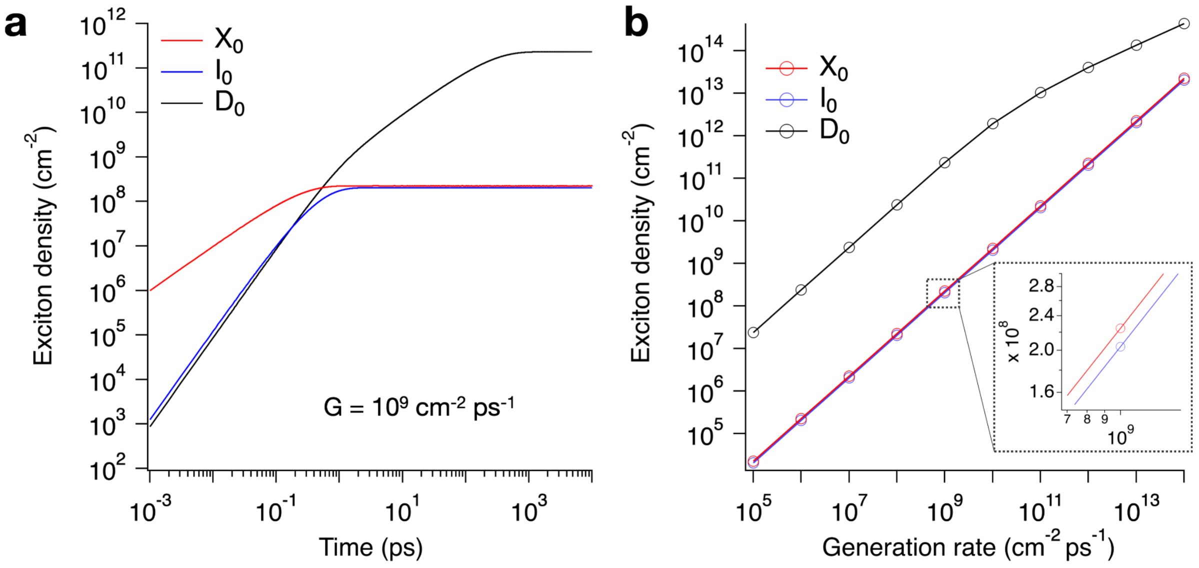

Figure S15: Calculations of exciton density. **a** - Dynamics of exciton populations for generation rate $G = 10^{9} cm^{-2} ps^{-1}$. **b** - Exciton density at the steady state as a function of the generation rate. The inset shows a zoom of the curves associated to the density of X^{0} and I^{0}.

We note that more sophisticated models should be developed to capture the full dynamics of carriers in W-based monolayers. The inclusion trions should not change qualitatively our conclusions because the relaxation processes of bright trionic states mostly occur among dark trionic states and have similar rates as the one of neutral excitons.[27] However, trions would introduce extra relaxation channels for the bright excitons and should be considered for a more accurate estimate. Moreover, the finite density of chiral phonons in the system could lead to different population dynamics at high generation rates. In the model, we have neglected the relaxation path towards the lower-energy momentum-forbidden $K\Lambda$ exciton because this interband scattering process in WS_2 has been found to be significant only in the presence of compressive strain. [30,31]

Further interaction mechanisms other than exchange interaction could potentially impact transport, including direct interaction and exciton-exciton annihilation. The direct interactions between spin-allowed *KK-KΛ* and *KK-KK'* excitons have been predicted to be attractive, while the direct interaction between *KK-KK* excitons is repulsive.[21] We can assume a similar dynamics for spin-forbidden excitons. However, the strength of these direct interactions is much weaker than exchange interactions. Therefore, in a first approximation, their role in the interaction-driven transport can be neglected. The impact of Auger recombination is considered by including exciton-exciton annihilation in the theoretical model used to calculate the exciton density. The results in Figure 5b and S15b show that, at high generation rates ($> 10^{11} cm^{-2} ps^{-1}$), the dark exciton density, and thus transport, is significantly affected by Auger recombination. However, in the range of generation rates used in our experiments ($\sim 10^{9} cm^{-2} ps^{-1}$), our calculations return a linear behavior suggesting that the effect of Auger recombination is weak.

9. Excitation, transport, and relaxation pathways for dark exciton

Figure 5c illustrates the transport and possible relaxation pathways for the dark exciton D^0. The long lifetime and high density of dark excitons favor the increase of the overall interaction energy of the dark exciton population at the excitation position. The exciton energy can be summarized as

$$E_X(x) = E_{BG}(x) - E_B + \Delta E(x)$$

where $E_{BG}(x)$ is the bandgap that depends on the spatial position due to the strain landscape engineered in the sample, E_B is the exciton binding energy that does not depends on the spatial coordinate (x) because strain only weakly affects it in the strain range investigated in this work,[32] $\Delta E(x)$ is the interaction energy. $\Delta E(x)$ is proportional to the exciton density $n(x)$ that follows the Gaussian laser profile used for excitation. The strong repulsive interaction at the excitation position provides initial momentum to excitons which diffuse away even towards locations of the sample with higher energy landscape. This latter case is used as an example in Fig. 5. The transported hot dark excitons can then thermalize toward the bottom of their energy dispersion via scattering with low energy Γ acoustic phonons.[6] From this state they can radiatively recombine in the form of a dark exciton D^0, dark trions D^-, phonon replica, T_1 or intervalley excitons I^0.

10. Supplementary experimental data for exciton diffusion

Figure S16 shows the results of further measurements of the uphill diffusion of dark excitons in locations with a similar energy landscape taken at different powers. In Fig. S16a, the excitation power is 150 µW, while in Fig. S16b is 100 µW. Dark excitons can diffuse across the full uphill energy landscape of 20 meV in the former case. However, in the latter case, the emission of dark excitons vanishes beyond a distance of ~ 4.5 µm, which corresponds approximately to 12 meV uphill diffusion. This behavior seems to indicate that uphill transport is now limited because the blue shift induced by dark exciton interaction cannot fully overcome the energy barrier. However, we cannot exclude that the signal from dark excitons gets too weak to be detected. Conducting measurements at lower power is challenging because the signal from diffused excitons decreases exponentially away from the excitation center.

In Figure S17, we present results for uphill diffusion measurements performed on a smaller energy landscape with a height of 4 meV that turns into a region with a “flat” energy landscape. These measurements are taken at a laser power of 100 µW. Figure S17b shows that bright excitons, trions, and biexciton complexes do not move uphill, consistent with our previous observations. However, dark excitons are able to overcome the energy energy barrier and reach the plateau at the end of the energy ramp. Although we observe strong emissions from dark excitons beyond the uphill region, the emission from the plateau does not provide any further information about the actual blueshift.

Figure S18 shows the diffusion across a flat and inhomogeneous region. In this experiment, D^0 and I^0 emerge from the bright trion shoulders only when the energy landscape changes by a few meV.

Surprisingly, in some uphill experiments, we observe a peak stemming from the neutral exciton and blue shifting as a function of the distance. The intensity of this peak is very dim and its energy is highlighted with yellow markers in Fig.16. Since the neutral exciton is not expected to have interaction and therefore not to diffuse uphill, we speculate that this emission could be ascribed to the spin and momentum forbidden dark exciton which has never been observed before. This very dark exciton, labeled DI^0, is composed of a hole in the valence band $K_{\uparrow}$ and the electron in the conduction band $K'_{\downarrow}$. DI^0 has very little probability to recombine optically as its phonon replicas are also dark. Interaction with Γ_5 phonons would lead to the transition $DI^0 \rightarrow I^0$, while interaction with K_3 or K_2 phonons to the transition $DI^0 \rightarrow D^0$. Interaction with K_1 phonon would result in the valley migration of the hole preserving its spin and it would give rise to another spin-forbidden transition. Therefore, the optical recombination of DI^0 would require scattering with multiple phononic modes that dramatically decrease its likelihood. The direct observation of this very dark mode in our experiments could be the result of perturbation-driven recombination due to disorder in the sample.[6,10]

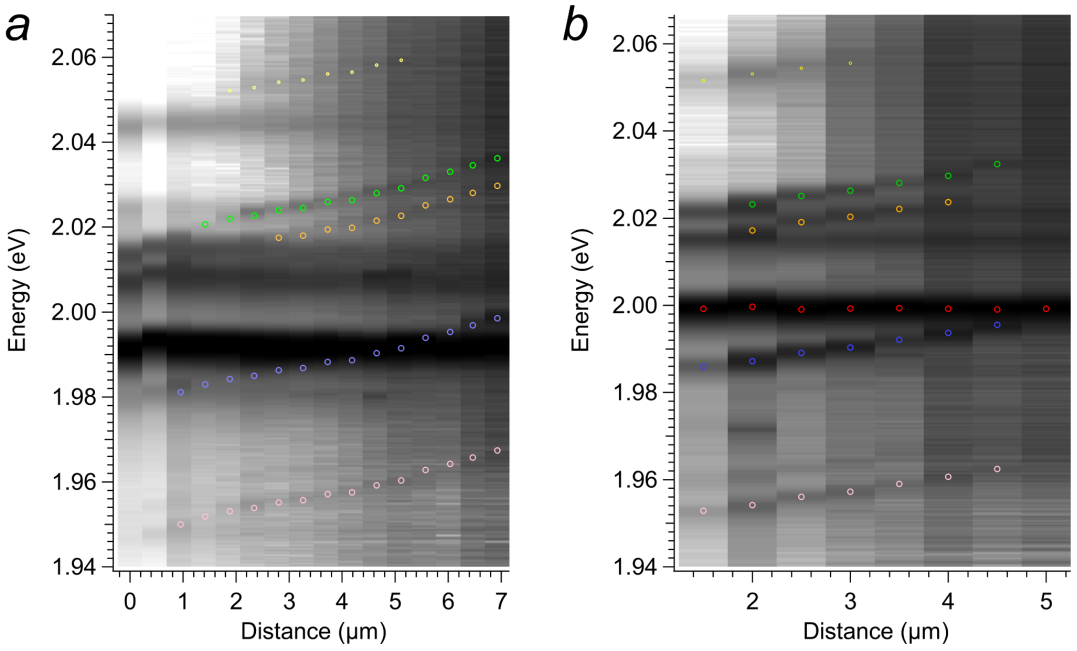

Figure S16: Uphill diffusion of excitons created at the bottom of a potential gradient and measured at increasing distances from the generation location. Experiments are performed in the same energy landscape, similar to the one of Fig.4 with different excitation power. (**a**) is 150 , (**b**) is 100 μW.

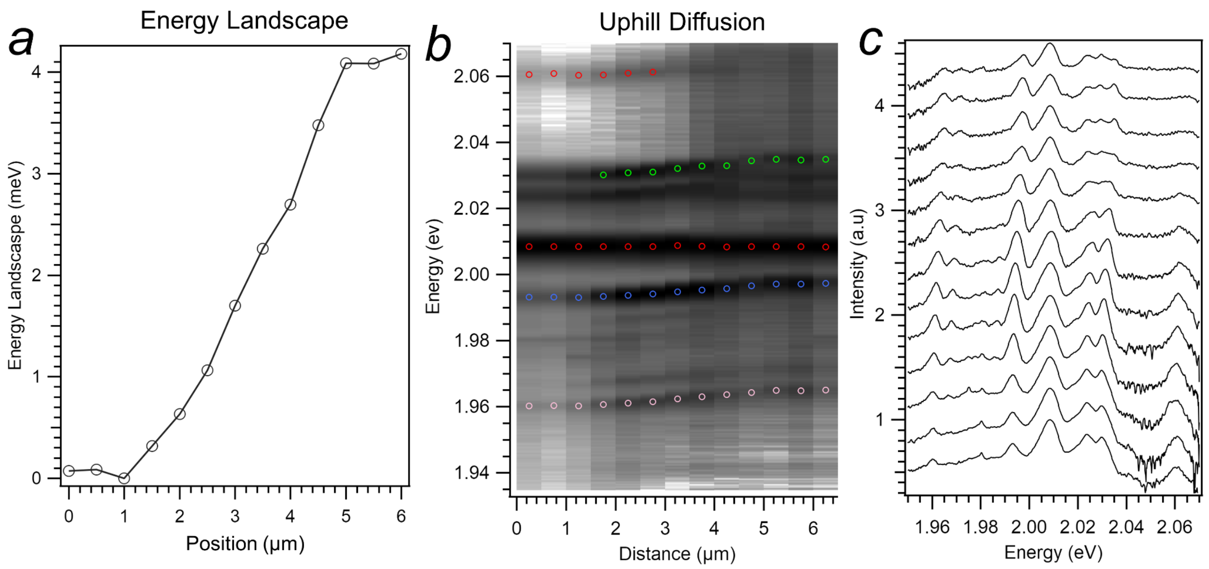

Figure S17: **a** - Uphill Energy Landscape of height 4 meV followed by flat energy landscape. This energy landscape is obtained by exciting and collecting the signal at the same position along a line on the sample. **b** - Diffusion measurement carried out in the energy landscape shown in **a**. This measurement is done by laser excitation at the initial position and collecting at different distances along the same line of the energy landscape. **c** - Raw data of uphill diffusion measurements in **b**.

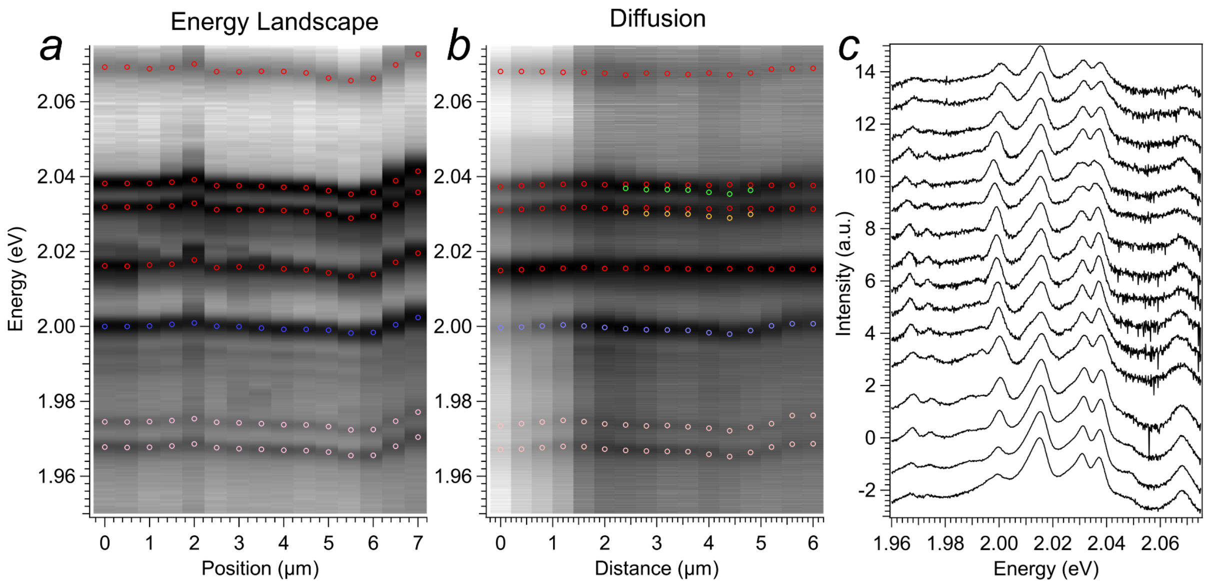

Figure S18: a - Non-uniform energy landscape that is initially flat, then slightly downhill and finally uphill. This energy landscape is measured by exciting and collecting the signal at the same position along a line on the sample. **b** - The diffusion measurement is carried out in the energy landscape shown in **a**. This measurement is done by exciting at the initial position and collecting at different distances along the same line of the energy landscape. **c** - Raw data of diffusion measurements in **b**.

REFERENCES

1. Zinkiewicz, M. *et al.* Excitonic Complexes in n-Doped WS2 Monolayer. *Nano Lett.* **21**, 2519–2525 (2021).
2. Zinkiewicz, M. *et al.* Neutral and charged dark excitons in monolayer WS2. *Nanoscale* **12**, 18153–18159 (2020).
3. Liu, E. *et al.* Valley-selective chiral phonon replicas of dark excitons and trions in monolayer WSe_2. *Phys. Rev. Research* **1**, 032007 (2019).
4. Li, Z. *et al.* Momentum-Dark Intervalley Exciton in Monolayer Tungsten Diselenide Brightened via Chiral Phonon. *ACS Nano* **13**, 14107–14113 (2019).
5. He, M. *et al.* Valley phonons and exciton complexes in a monolayer semiconductor. *Nat. Commun.* **11**, 618 (2020).
6. Dery, H. & Song, Y. Polarization analysis of excitons in monolayer and bilayer transition-metal dichalcogenides. *Phys. Rev. B Condens. Matter* **92**, 125431 (2015).
7. Li, P. *et al.* Intervalley electron-hole exchange interaction and impurity-assisted recombination of indirect excitons in WS_2 and WSe_2 monolayers. *Phys. Rev. B Condens. Matter* **106**, 085414 (2022).
8. Steinhoff, A. *et al.* Biexciton fine structure in monolayer transition metal dichalcogenides. *Nat. Phys.* **14**, 1199–1204 (2018).
9. Wang, G. *et al.* In-Plane Propagation of Light in Transition Metal Dichalcogenide Monolayers: Optical Selection Rules. *Phys. Rev. Lett.* **119**, 047401 (2017).
10. Slobodeniuk, A. O. & Basko, D. M. Spin–flip processes and radiative decay of dark intravalley excitons in transition metal dichalcogenide monolayers. *2D Mater.* **3**, 035009 (2016).
11. Li, Z. *et al.* Direct Observation of Gate-Tunable Dark Trions in Monolayer WSe2. *Nano Lett.* **19**, 6886–6893 (2019).

12. Liu, E. *et al.* Multipath Optical Recombination of Intervalley Dark Excitons and Trions in Monolayer WSe_2. *Phys. Rev. Lett.* **124**, 196802 (2020).
13. Danovich, M., Zólyomi, V. & Fal'ko, V. I. Dark trions and biexcitons in WS2 and WSe2 made bright by e-e scattering. *Sci. Rep.* **7**, 1–5 (2017).
14. Steinhoff, A., Rösner, M., Jahnke, F., Wehling, T. O. & Gies, C. Influence of excited carriers on the optical and electronic properties of MoS_2. *Nano Lett.* **14**, 3743–3748 (2014).
15. Sie, E. J. *et al.* Observation of Exciton Redshift-Blueshift Crossover in Monolayer WS2. *Nano Lett.* **17**, 4210–4216 (2017).
16. Sie, E. J., Frenzel, A. J., Lee, Y.-H., Kong, J. & Gedik, N. Intervalley biexcitons and many-body effects in monolayer MoS_2. *Phys. Rev. B Condens. Matter* **92**, 125417 (2015).
17. Jauregui, L. A. *et al.* Electrical control of interlayer exciton dynamics in atomically thin heterostructures. *Science* **366**, 870–875 (2019).
18. Wang, G. *et al.* Valley dynamics probed through charged and neutral exciton emission in monolayerWSe2. *Physical Review B* vol. 90 Preprint at https://doi.org/10.1103/physrevb.90.075413 (2014).
19. Robert, C. *et al.* Fine structure and lifetime of dark excitons in transition metal dichalcogenide monolayers. *Phys. Rev. B Condens. Matter* **96**, 155423 (2017).
20. Shahnazaryan, V., Iorsh, I., Shelykh, I. A. & Kyriienko, O. Exciton-exciton interaction in transition-metal dichalcogenide monolayers. *Phys. Rev. B Condens. Matter* **96**, 115409 (2017).
21. Erkensten, D., Brem, S. & Malic, E. Exciton-exciton interaction in transition metal dichalcogenide monolayers and van der Waals heterostructures. *Physical Review B* vol. 103 Preprint at https://doi.org/10.1103/physrevb.103.045426 (2021).

22. (Li P. *et al.* Intervalley electron-hole exchange interaction and impurity-assisted recombination of indirect excitons in WS2 and WSe2 monolayers. *Phys. Rev. B Condens. Matter* **106**, 085414 (2022).
23. Ye, Z. *et al.* Probing excitonic dark states in single-layer tungsten disulphide. *Nature* **513**, 214–218 (2014).
24. Robert, C. *et al.* Measurement of the spin-forbidden dark excitons in MoS2 and MoSe2 monolayers. *Nat. Commun.* **11**, 1–8 (2020).
25. Chernikov, A. *et al.* Exciton Binding Energy and Nonhydrogenic Rydberg Series in Monolayer WS2. *Phys. Rev. Lett.* **113**, 076802 (2014).
26. Stier, A. V., McCreary, K. M., Jonker, B. T., Kono, J. & Crooker, S. A. Exciton diamagnetic shifts and valley Zeeman effects in monolayer WS2 and MoS2 to 65 Tesla. *Nat. Commun.* **7**, 1–8 (2016).
27. Yang, M. *et al.* Relaxation and darkening of excitonic complexes in electrostatically doped monolayer WSe2 : Roles of exciton-electron and trion-electron interactions. *Physical Review B* vol. 105 Preprint at https://doi.org/10.1103/physrevb.105.085302 (2022).
28. Hoshi, Y. *et al.* Suppression of exciton-exciton annihilation in tungsten disulfide monolayers encapsulated by hexagonal boron nitrides. *Phys. Rev. B Condens. Matter* **95**, 241403 (2017).
29. Zipfel, J. *et al.* Exciton diffusion in monolayer semiconductors with suppressed disorder. *Phys. Rev. B Condens. Matter* **101**, 115430 (2020).
30. Feierabend, M., Khatibi, Z., Berghäuser, G. & Malic, E. Dark exciton based strain sensing in tungsten-based transition metal dichalcogenides. *Phys. Rev. B Condens. Matter* **99**, 195454 (2019).
31. Chand, S. B. *et al.* Visualization of Dark Excitons in Semiconductor Monolayers for

High-Sensitivity Strain Sensing. *Nano Lett.* **22**, 3087–3094 (2022).

32. Aslan, B., Deng, M. & Heinz, T. F. Strain tuning of excitons in monolayer WSe_2. *Phys. Rev. B Condens. Matter* **98**, 115308 (2018).
